# Focal nodular hyperplasia combined with hepatocellular carcinoma with bone metastasis: a case report and literature review

**DOI:** 10.3389/fonc.2025.1671301

**Published:** 2025-10-24

**Authors:** Yuliang Zhang, Xiyue Deng, Kaiyue Cao, Yang Li

**Affiliations:** ^1^ Department of Hepatobiliary Pancreatic Surgery, Tianjin First Central Hospital, School of Medicine, Nankai University, Tianjin, China; ^2^ Department of Pathology, Tianjin First Central Hospital, School of Medicine, Nankai University, Tianjin, China

**Keywords:** liver, focal nodular hyperplasia, hepatocellular carcinoma, bone metastasis, surgery

## Abstract

Focal nodular hyperplasia (FNH), the second most common benign liver lesion, is widely regarded as having no malignant potential. This study reports a 29-year-old male who was admitted to the hospital due to a space-occupying liver lesion accompanied by low back pain. Abdominal contrast-enhanced computed tomography (CE-CT) suggested FNH. The patient underwent surgical treatment, and postoperative pathological results revealed both FNH and hepatocellular carcinoma (HCC). The patient still experienced low back pain symptoms after surgery. He was later confirmed to have bone metastasis (BM) and received radiotherapy. Cases combining FNH and HCC are extremely rare, particularly when accompanied by metastasis. This study aims to report a case of FNH combined with HCC and accompanied by BM, and to review its treatment process in detail.

## Case report

**Figure 1:**
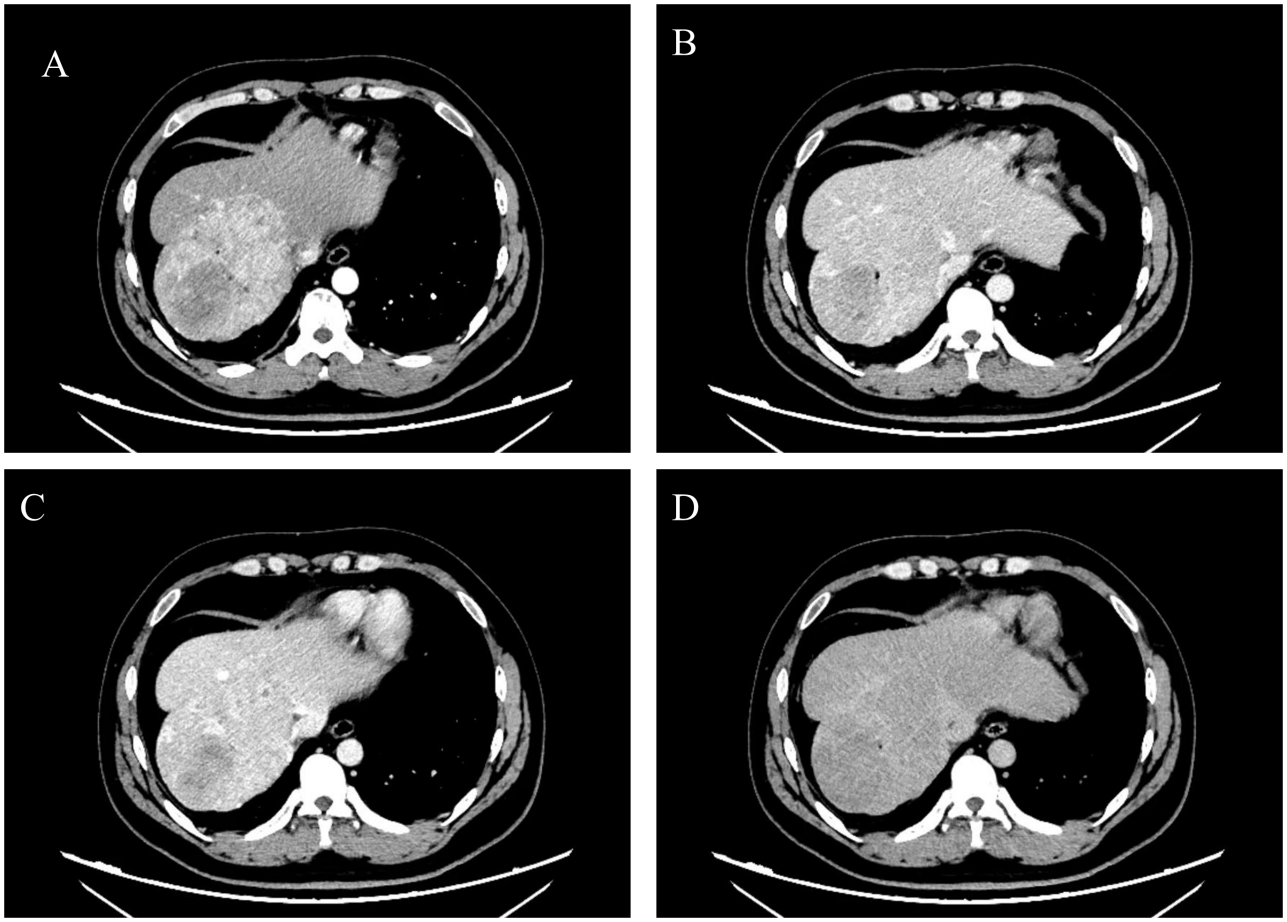
Preoperative abdominal CE-CT scan revealed a space-occupying lesion in liver segments S7-8, with significant heterogeneous enhancement during the arterial phase **(A)**, reduced enhancement during the venous phase **(B)** and portal phase **(C)**, and a heterogeneous low-density shadow in the delayed phase **(D)**. The presented cross-sections are all at the level of the T10 vertebral body.

A 29-year-old male patient was admitted to our hospital in August 2024 due to “space-occupying liver lesion for 10 years and intermittent low back pain for 1 month”. The liver lesion was initially detected during a physical examination 10 years ago and was considered to be Focal nodular hyperplasia (FNH). The patient underwent regular abdominal ultrasound examinations, which revealed slow progression of the lesion. No specific diagnostic workup or treatment was administered during this period. 1 month prior to admission, the patient developed intermittent right-sided low back pain. After presentation to our hospital, abdominal contrast-enhanced computed tomography (CE-CT) demonstrated a large mass measuring approximately 8.5×11.4cm in the right hepatic lobe. Imaging features of the mass included obvious heterogeneous enhancement in the arterial phase, decreased enhancement in the venous phase, and heterogeneous hypodensity in the delayed phase, which was considered as FNH (**Figure 1**). The main differential diagnosis included diseases such as HCC, intrahepatic cholangiocarcinoma (ICC), hepatic hemangioma, and secondary malignant liver tumors. Laboratory tests revealed normal complete blood count (WBC: 7.02×10^9^/L, HGB: 155g/L), normal liver function (ALT: 29U/L, AST: 2.2U/L, TBIL: 17.83μmol/L, ALB: 48.8g/L), normal kidney function(CREA: 56.2μmol/L), normal coagulation function (PT: 11.8s, INR: 0.94). The alpha-fetoprotein (AFP) level was 1.38ng/mL, and serological tests for viral hepatitis were all negative. The patient’s liver function was classified as Child-Pugh Class A. The patient denied any history of chronic diseases, viral hepatitis, or relevant familial genetic disorders. Abdominal examination showed no significant abnormalities. In addition, the patient was in good health and had not received any medication or hormonal therapy.

**Figure 2:**
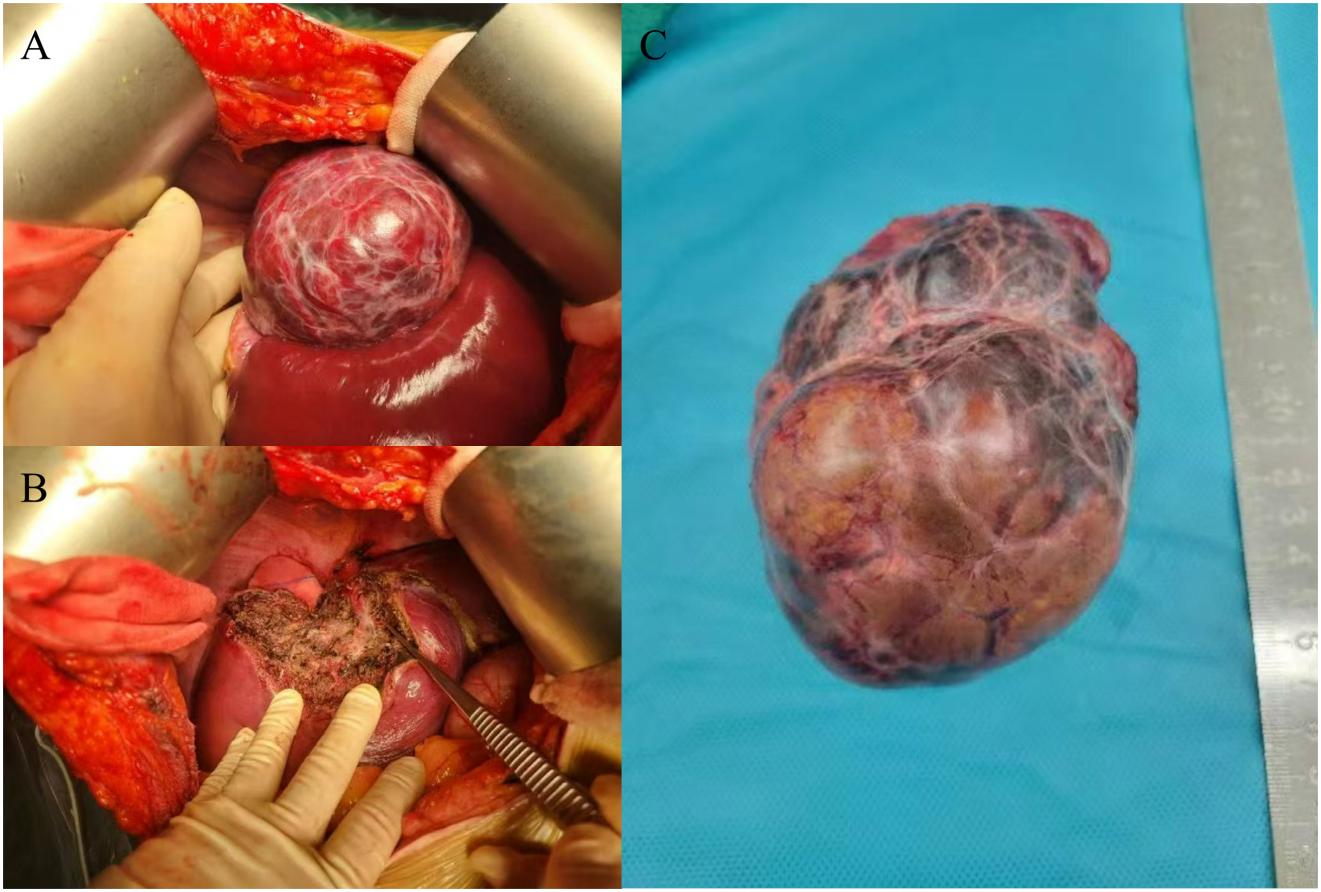
Intraoperative findings and postoperative specimens. Completely exposed tumor and part of the liver **(A)**, surgical field after complete tumor resection **(B)**, tumor specimen **(C)**.

**Figure 3:**
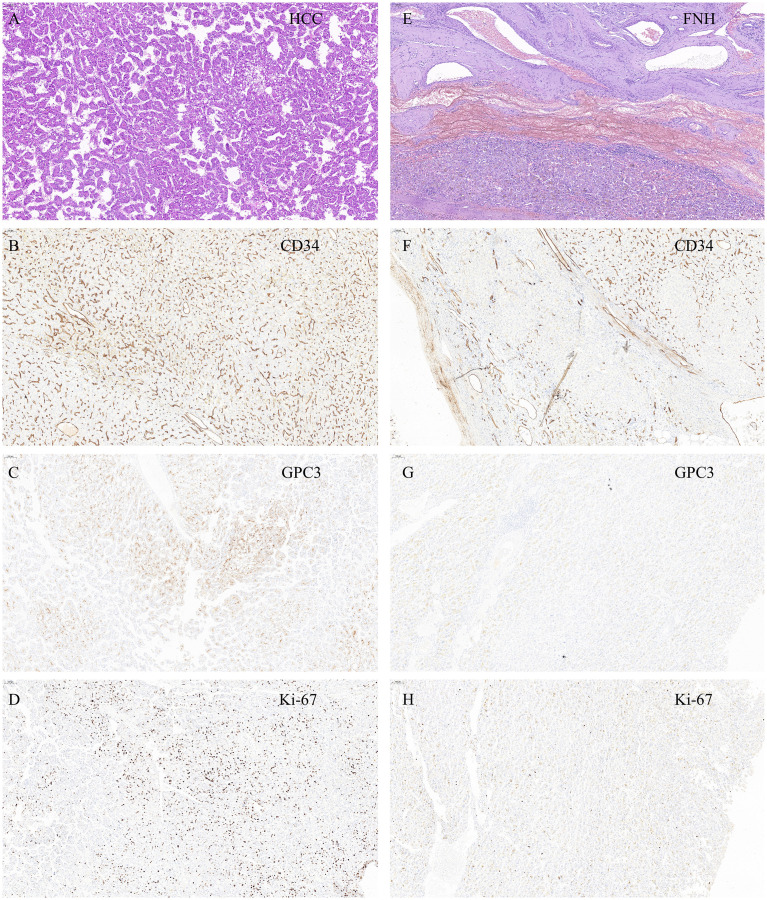
**(A)** H&E staining. The hepatic plate structure of the liver tissue is disrupted, arranged thin trabecular or pseudoglandular patterns, with mild cellular atypia. findings suggestive of HCC. **(B)** HCC-CD34 staining shows a dense microvascular structure, suggesting hepatic sinusoidal capillarization. **(C)** HCC-GPC3 staining shows that most of the tumor cells are positive. **(D)** HCC-Ki-67 staining shows a high proliferation index of tumor cells, about 40% -50% positivity in dense areas. **(E)** H&E staining. The hepatic plate structure of the liver tissue is relatively intact, with no cellular atypia. Multiple thick walled arterial vessels and proliferated small bile ducts are observed at the periphery. findings suggestive of FNH. **(F)** FNH-CD34 staining shows no hepatic sinusoidal capillarization in the liver tissue. **(G)** FNH-GPC3 staining is negative. **(H)** FNH-Ki-67 staining shows a low cell proliferation index of cells, about 1% -2%. All pathological images were captured at a magnification of 100× and stained with a Roche staining machine.

After admission, we communicated with the patient. Due to the large tumor size and painful symptoms, the patient expressed a strong desire for surgical intervention. Following the exclusion of surgical contraindications based on the preoperative evaluation, the patient subsequently underwent surgery. During the operation, the liver was observed to be of normal size with a smooth surface and soft texture. A large mass protruding from the liver surface was observed in segments VII and VIII. The mass was completely resected surgically (**Figure 2**). Gross pathological findings of the postoperative specimen revealed a piece of grayish-brown nodular tissue, measuring approximately 18×8×6cm. The cut surface demonstrated a grayish-yellow nodule, measuring up to 7 cm in greatest dimension, which was relatively soft in consistency, well-demarcated from the surrounding parenchyma, and abutted the capsule. The cut surfaces of the remaining liver tissue were grayish-brown with an irregular nodularity. Microscopic examination revealed a HCC, approximately 7cm in diameter, which was well to moderately differentiated with trabecular and pseudoglandular patterns. There was no microvascular invasion (MVI = 0), and all surgical margins were negative. Immunohistochemistry staining showed GPC3(+), CD34 (indicating dense microvessels), and the Ki-67 proliferative index was approximately 40%-50% in the dense cell areas. The background liver parenchyma was subdivided into nodules by fibrous septa, which contained thick-walled arteries and proliferative bile ductules. No significant hepatocyte atypia was observed. In conjunction with the clinical history and imaging findings, these features were diagnostic of FNH. Immunohistochemistry staining showed CD34 (indicating dense microvessels) and CK19 (positive in small bile ducts) (**Figure 3**).

**Figure 4:**
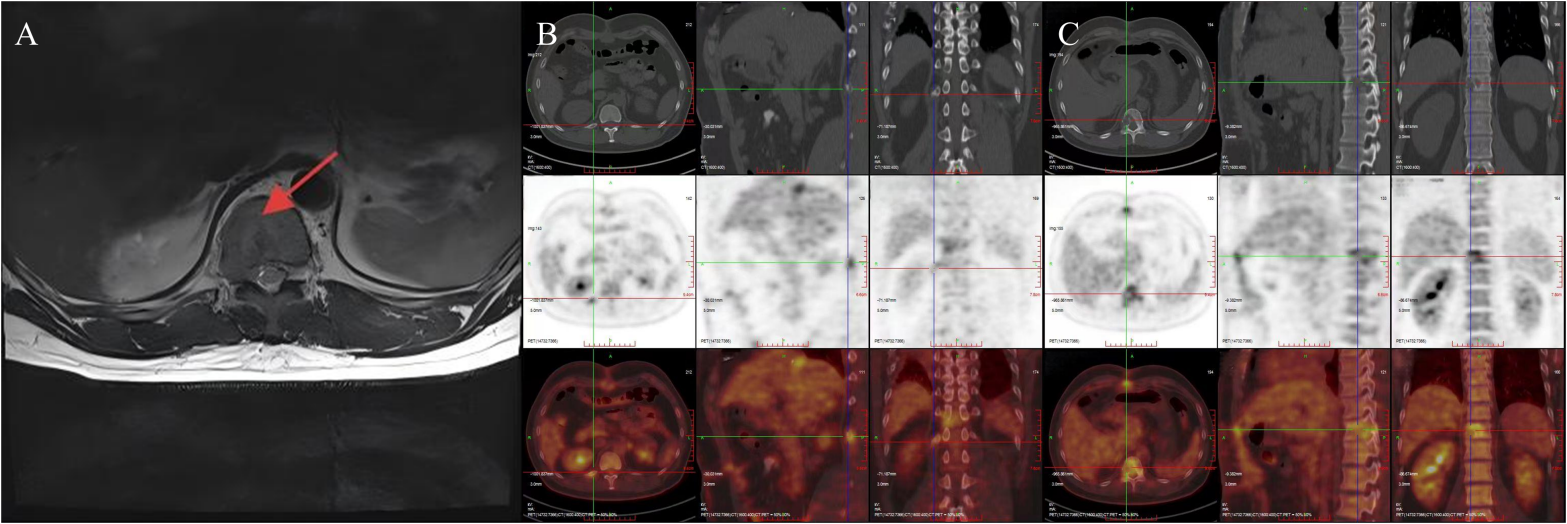
MRI indicates bone destruction, suggesting bone metastasis **(A)**; PET-CT reveals irregular morphology of the right 12th rib, 11th thoracic vertebra, and right vertebral arch, accompanied by bone destruction and increased metabolism, suggesting metastasis **(B, C)**.

The patient recovered well postoperatively and was discharged uneventfully. However, during follow-up, it was noted that he continued to experience persistent right-sided low back pain, which progressively interfered with his daily activities, work, and sleep. A lumbar spine magnetic resonance imaging(MRI) performed in October 2024 revealed osteolytic destruction of the right 12th rib, the T11 vertebral body, and the right pedicle, accompanied by an epidural mass, malignant metastasis was considered (**Figure 4A**). Positron Emission Tomography-Computed Tomography (PET-CT) also showed irregular morphology of the right 12th rib, the T11 vertebral body and the right pedicle, accompanied by bone destruction, increased metabolism, considered metastasis. No other abnormalities were detected (**Figures 4B, C**). Then the patient received stereotactic radiotherapy for bone metastasis(BM), with the radiation dose specified as follows: 95% Planning Target Volume(PTV) 1: 45Gy in 10 fractions(4.5Gy per fraction); 95% PTV2: 40Gy in 10 fractions(4Gy per fraction), totaling 20 fractions. After the completion of radiotherapy, we performed a prophylactic transcatheter arterial chemoembolization(TACE) for the patient. During the procedure, selective hepatic angiography was conducted, and the results showed no abnormal enhancement in the liver. Subsequently, the patient received regular follow-up visits at the outpatient clinic. Both AFP testing and abdominal imaging examinations revealed no evidence of tumor recurrence. The patient’s pain symptoms resolved, and he resumed normal work and daily life.

## Discussion

FNH is a proliferative response triggered by vascular malformations during hepatic development. It ranks as the second most common benign hepatic lesion, with a prevalence of 0.3% to 3% in the general population (1, 2). This condition is more prevalent in women of childbearing age, typically presenting as a single lesion without significant clinical symptoms. Most cases are incidentally detected during routine physical examinations, whereas a small subset of patients may experience symptoms such as chronic abdominal pain, abdominal masses, nausea, dyspepsia, and early satiety (1, 3). HCC ranks as the sixth most common malignancy worldwide and the fourth leading cause of cancer-related deaths. As global population aging intensifies and the prevalence of chronic liver disease rises, the disease burden associated with HCC continues to grow progressively (4, 5). In 2019, there were approximately 747,000 cases of HCC globally, representing a 70% increase since 1990. Additionally, HCC was responsible for an estimated 480,000 deaths that year (4). Although several potentially curative treatment options are currently available (e.g., liver transplantation, surgical resection, and thermal ablation), the insidious onset of HCC means that fewer than 30% of patients are eligible for such curative therapies at the time of initial diagnosis (6, 7). Previous studies have reported that even with systemic therapy, the median survival time of patients with symptomatic advanced-stage HCC remains very short, at only 1.0 to 1.5 years (8). Compared with other common malignant tumors, HCC carries a higher risk of metastasis (9). Among these metastatic sites, the lungs are the most common, followed by lymph nodes, bones, and the adrenal glands (9, 10). Approximately 16.1% to 38.5% of HCC patients present with BM at initial diagnosis, and 11.7% of HCC patients who undergo radical resection subsequently develop BM (11). HCC related BM is predominantly osteolytic, characterized by patchy or erosive reductions in bone density. However, it may also present as osteoblastic metastasis, accompanied by the formation of expansive soft tissue masses (12). Currently, extrahepatic metastasis is a common characteristic of advanced HCC, and HCC with BM further indicates stronger invasiveness of the disease and a poorer prognosis (13). HCC with BM is typically associated with severe pain, pathological fractures, and other nerve compression-related conditions (14). According to reports, the median survival time of HCC patients with BM is only 4.6 months (13).

FNH is generally regarded as having no malignant potential, however, a very small number of cases involving the co-occurrence of FNH and HCC have been reported (**Table 1**, **Supplementary Table 1**). The case reports included in this study all adhered to the requirements of the CARE guidelines. A total of 20 patients were reviewed, including 4 male patients and 16 female patients, with a mean age of 44.8 ± 20.1 years. Among these patients, the lesions were distributed adjacently in 9 cases, whereas in 11 cases, one lesion was wrapped around the other. Except for one patient who developed lung metastasis, no metastatic lesions were detected in the remaining patients. Additionally, most patients underwent surgical intervention. In contrast to previous studies, this study is the first to report BM in a patient with FNH combined with HCC.

**Table 1:** Summary of case reports on FNH combined with HCC.

First author	Year	Number	Age /years	Sex	Diameter (cm)	Positional relationship	Metastasis	Treatment
Saul SH (15)	1987	1	19	Female	9.0x8.5x5.0	Adjacent	No	Surgery
Davidson BR (16)	1990	1	16	Male	6.0	Contain	No	Surgery
Saxena R (17)	1994	1	14	Female	5.0	Contain	No	–
Chen TC (18)	2001	1	65	Female	20×18×12	Adjacent	No	Surgery
Coopersmith CM (19)	2002	1	43	Female	HCC: 7×6.5×3.5 FNH: 0.3-3.0	Adjacent	Yes	Chemotherapy
Cucchetti A (20)	2003	1	55	Female	NR	Adjacent	No	Surgery
Zhang SH (21)	2004	1	56	Male	HCC: 3×2.5×2.0 FNH: 2×1.5×1.5	Adjacent	No	Surgery
Imkie M (22)	2005	2	27	Female	HCC: 6.0 FNH: 20.0	Adjacent	No	Surgery
			45	Female	HCC: NR FNH: 14.0	Adjacent	No	Surgery
Langrehr JM (23)	2006	2	46	Female	HCC: 5.0 FNH: 12.0	Contain	No	Surgery
			50	Female	HCC: 3.0 FNH: 14.0	Contain	No	Surgery
Petsas T (24)	2006	1	23	Female	HCC: 2.1×1.8×1.0 FNH: 9.0×6.0×5.0 4.5×3.5×2.0	Contain	No	Surgery
Sotiropoulos GC (25)	2008	1	31	Female	HCC: 7.0 FNH: 15.0	Adjacent	No	Surgery; Chemotherapy
Morise Z (26)	2009	1	59	Male	HCC: 0.9 FNH: 2.0	Contain	No	Surgery
Haubert L (27)	2010	1	86	Female	8.5×5.5×4.8	Contain	No	Surgery
Scheuermann U (28)	2012	1	38	Female	NR	–	No	Surgery
Koea JB (29)	2014	2	34	Female	NR	Contain	No	Surgery
			45	Male	NR	Contain	No	Surgery
Ercan C (30)	2022	1	74	Female	2.9	Adjacent	No	Surgery
Udquim KT (31)	2024	1	70+	Female	13.0x13.0x9.0	Contain	No	Surgery

Number, number of patients. NR, not reported. -, not applicable.

We hypothesize that the simultaneous occurrence of FNH and HCC may be attributed to the following two reasons. Firstly, FNH and HCC share common risk factors, including hepatic vascular abnormalities, a history of liver disease, and hormonal influences. Currently, it is widely accepted that FNH originates from vascular malformations, which can result in excessive blood perfusion and subsequently induce secondary proliferation/regeneration reactions in the hepatic parenchyma (32). The growth and metastasis of HCC are thought to depend on neovascularization. Recent studies have demonstrated that angiogenesis mediated by vascular endothelial cells is a key component of the tumor microenvironment(TME) that drives the progression of HCC (33). The presence of abnormal hepatic vasculature increase the risk of their concurrent occurrence. It is currently established that individuals with Budd-Chiari syndrome (BCS), congenital absence of the portal vein (CAPV), or cirrhosis have a higher risk of developing either FNH or HCC (34–38). The essence of the aforementioned increased risk is closely linked to vascular abnormalities. A study investigating the clinicopathological features of FNH-like hepatic nodules revealed that vascular alterations in cirrhosis may play a critical role in the pathogenesis of such nodules (39). For HCC, there is ample evidence to suggest that liver cirrhosis is a key risk factor, and the reasons for this increased risk include liver cirrhosis causing abnormal hepatic vasculature (40–43). Meanwhile, the persistent hepatic inflammatory response in cirrhosis can induce hepatocellular injury and impair the hepatic repair process, further increasing the risk of HCC development, building on the foundational role of vascular abnormalities in providing nutritional support and metastatic pathways (44). In addition, a sustained inflammatory response can upregulate the expression of angiogenesis related factors such as vascular endothelial growth factor (VEGF), which also contributes to aberrant angiogenesis. As is well established, the liver is a hormone sensitive organ that expresses both estrogen and androgen receptors. Several studies have indicated that long-term use of oral contraceptives (OCs) may potentially induce the development of benign tumors(including FNH) and malignant tumors(including HCC) (45, 46). Research suggests that hormones may act on hepatocytes by regulating cell proliferation and differentiation, thereby increasing the probability of concurrent HCC and FNH (45). Secondly, from a genetic standpoint, previous studies have confirmed that a certain correlation may exist between FNH and HCC (30). Ercan C et al. were the first to report in 2022 that FNH may possess the potential for malignant transformation and progression to HCC in extremely rare cases. In this study, a patient with hepatic nodules underwent surgical intervention, and postoperative pathological findings revealed that the nodules consisted of FNH and two distinct HCC components(Edmondson grade 1 and grade 2) (30). Researchers conducted whole exome sequencing on FNH component and two HCC components, and the results showed that 80 non-synonymous mutations(such as *ATG5*, *ANGPT1*, *HNRNPA2B1*, *CBL*) were found to coexist among the three, with HCC specific mutations including *MYCN* and *MAP2K4*; In addition, the telomerase reverse transcriptase gene(*TERT*) promoter regions of all three carry the hotspot mutation −c.124 C > T (30). This indicates that the three may have a common origin. No driver gene somatic mutations commonly found in HCC, such as *CTNNB1*, *TP53*, *AXIN1*, or *ARIDA1*, were detected among the three (29). Based on this, it is speculated that FNH related HCC may have a unique pathogenesis. Subsequent clonal analysis showed that all lesions were composed of multiple clones, all of which had a clonal cell population containing a set of mutations(CBL, ANGPT1, and ATG5), and the clones carrying *MYCN* and *MAP2K4* mutations in HCC did not appear in FNH (30). Clonal analysis confirmed the clonal relationship between FNH and HCC, and suggested that FNH may progress to HCC through clonal selection or accumulation of new mutations, although further evidence is needed to support this conclusion in the future.

FNH and HCC are prone to misdiagnosis or missed diagnosis in clinical practice, which may result in patients missing the optimal treatment window. The main reason is associated with the overlapping imaging features and atypical laboratory indicators between the two conditions. Firstly, from an imaging standpoint, typical FNH can be reliably diagnosed through the comparison of CT or MRI findings. However, atypical FNH may exhibit less intense enhancement, absence of a central scar, and pseudocapsular enhancement on delayed phase images, as well as the presence of hemorrhage, calcification, or necrosis. These features render it difficult to distinguish the imaging features of atypical FNH from those of HCC (47–49). Secondly, in most cases, AFP serves as a key tumor marker for diagnosing HCC. However, in some patients with concurrent FNH and HCC, AFP levels may remain within the normal range, failing to provide effective diagnostic cues and thereby increasing diagnostic difficulty (23). Furthermore, other routine liver function parameters(such as transaminase, bilirubin, and coagulation factors, etc.) may also be within the normal range in patients with concurrent FNH and HCC, alternatively, the changes in these parameters may lack significant discriminatory value (23, 50). In this study, given that the patient was relatively young, had no history of hepatitis, and tested negative for AFP, we initially assessed a low likelihood of HCC during differential diagnosis. However, the final pathological findings confirmed the presence of HCC components in the tumor. Upon retrospective review of this case, we noted that the T11 vertebral body of the patient’s abdominal CE-CT revealed patchy hypodense shadows, an indication that the patient may have already had BM before receiving surgical treatment. Additionally, the patient’s lower back pain was likely attributable to BM rather than local symptoms of the tumor. Owing to the atypical imaging features of this lesion on abdominal CE-CT, it may be necessary to further perform abdominal contrast-enhanced MRI or other supplementary examinations preoperatively to confirm the diagnosis. This represents a key oversight in our case management. Therefore, we aim to draw attention to this issue for clinical physicians: when managing patients with hepatic masses, particularly those with tumors located adjacent to bony structures(e.g., the spine or ribs), who present with pain in the trunk or extremities, clinicians should remain vigilant for the possibility of BM. In addition, in cases where FNH is considered a potential diagnosis but abdominal CE-CT findings are atypical, contrast-enhanced MRI should be recommended to further clarify the diagnosis.

Beyond being the first reported case of BM in a patient with concurrent FNH and HCC, this study has several limitations that merit attention. Firstly, while the discussion section addresses multiple possible mechanisms underlying the concurrent occurrence of FNH and HCC, the specific genetic driver most likely associated with this particular case remains unconfirmed. Second, the overall follow-up duration for the patient is still relatively short, and long-term monitoring of the patient’s condition will be necessary in subsequent periods. Third, most of the included case reports lack racial demographic data, making it difficult to clarify racial differences. Future research should delve deeper to clarify the pathogenesis of FNH combined with HCC, particularly at the genetic level.

Currently, follow-up monitoring is recommended for patients with FNH, and surgical intervention is typically reserved for cases where patients present with overt symptoms, lesion enlargement (≥5cm), or rapid lesion progression during follow-up. Against this backdrop, it is particularly important to be vigilant about the development of HCC in FNH patients undergoing follow-up, and how to achieve accurate differentiation between the two conditions remains one of the key challenges in contemporary liver disease management.

## Data Availability

The raw data supporting the conclusions of this article will be made available by the authors, without undue reservation.
