# Supplementary material for: Focal nodular hyperplasia combined with hepatocellular carcinoma with bone metastasis: a case report and literature review
Source: Front Oncol. 2025 Oct 24;15:1671301. doi: 10.3389/fonc.2025.1671301 (PMC12591948; doi:10.3389/fonc.2025.1671301)
Supplement: Supplementary file 1 [file DataSheet1.pdf]

Supplementary table 1. Inclusion in case reports quality assessment.

| Topic | | Case reports | | | | | | | | | | | | | | | | |
|---|---|---|---|---|---|---|---|---|---|---|---|---|---|---|---|---|---|---|
| | | (15) | (16) | (17) | (18) | (19) | (20) | (21) | (22) | (23) | (24) | (25) | (26) | (27) | (28) | (29) | (30) | (31) |
| Title | | | | | | | | | | | | | | | | | | |
| 1 | The diagnosis or intervention of primary focus followed by the words "case report" | | | | √ | | √ | √ | √ | | | | | | | | √ | √ |
| Key Words | | | | | | | | | | | | | | | | | | |
| 2 | 2 to 5 key words that identify diagnoses or interventions in this case report, including "case report" | | | √ | √ | √ | √ | √ | √ | √ | √ | | | | | | √ | √ |
| Abstract (no references) | | | | | | | | | | | | | | | | | | |
| 3a | Introduction: What is unique about this case and what does it add to the scientific literature? | √ | | √ | √ | √ | √ | √ | √ | √ | √ | | √ | √ | √ | √ | √ | √ |
| 3b | Main symptoms and/or important clinical findings | | | √ | √ | √ | √ | √ | √ | √ | √ | | √ | | √ | | √ | √ |
| 3c | The main diagnoses, therapeutic interventions, and outcomes | | | √ | √ | √ | √ | | √ | √ | | | √ | √ | √ | √ | √ | √ |
| 3d | Conclusion—What is the main "take-away" lesson(s) from this case? | √ | | √ | √ | √ | √ | √ | √ | √ | √ | | √ | √ | √ | √ | √ | √ |

| | | | | | | | | | | | | | | | | | | |
|---|---|---|---|---|---|---|---|---|---|---|---|---|---|---|---|---|---|---|
| Introduction | | | | | | | | | | | | | | | | | | |
| 4 | One or two paragraphs summarizing why this case is unique (may include references) . | √ | √ | √ | √ | √ | √ | √ | √ | √ | √ | √ | √ | √ | √ | √ | √ | √ |
| Patient information | | | | | | | | | | | | | | | | | | |
| 5a | De-identified patient specific information | √ | √ | √ | √ | √ | √ | √ | √ | √ | √ | √ | √ | √ | √ | √ | √ | √ |
| 5b | Primary concerns and symptoms of the patient | √ | √ | √ | √ | √ | √ | √ | √ | √ | √ | √ | √ | √ | √ | √ | √ | √ |
| 5c | Medical, family, and psycho-social history including relevant genetic information | | | | √ | √ | √ | √ | √ | √ | √ | | √ | | | | √ | √ |
| 5d | Relevant past interventions with outcomes | √ | √ | | | √ | √ | | | | √ | √ | | | √ | | √ | √ |
| Clinical Findings | | | | | | | | | | | | | | | | | | |
| 6 | Describe significant physical examination (PE) and important clinical findings | √ | √ | √ | √ | √ | √ | √ | √ | √ | √ | √ | √ | √ | √ | √ | √ | √ |
| Timeline | | | | | | | | | | | | | | | | | | |
| 7 | Historical and current information from this episode of care organized as a timeline | | √ | | √ | √ | √ | | √ | √ | √ | √ | √ | √ | √ | √ | √ | √ |
| Diagnostic Assessment | | | | | | | | | | | | | | | | | | |
| 8a | Diagnostic testing (such as PE, laboratory testing, imaging, surveys) | √ | √ | √ | √ | √ | √ | √ | √ | √ | √ | √ | √ | √ | √ | √ | √ | √ |

| | | | | | | | | | | | | | | | | | | |
|---|---|---|---|---|---|---|---|---|---|---|---|---|---|---|---|---|---|---|
| 8b | Diagnostic challenges (such as access to testing, financial, or cultural) | | | | | | | | | √ | √ | | | √ | √ | | √ | |
| 8c | Diagnosis (including other diagnoses considered) | √ | √ | √ | √ | √ | √ | √ | √ | √ | √ | √ | √ | √ | √ | √ | √ | √ |
| 8d | Prognosis (such as staging in oncology) where applicable | | | √ | √ | √ | √ | √ | √ | √ | √ | √ | | √ | √ | √ | √ | √ |
| Therapeutic intervention | | | | | | | | | | | | | | | | | | |
| 9a | Types of therapeutic intervention (such as pharmacologic, surgical, preventive, self-care) | √ | √ | | √ | √ | √ | √ | √ | √ | √ | √ | √ | √ | √ | √ | √ | √ |
| 9b | Administration of therapeutic intervention (such as dosage, strength, duration) | | | | √ | √ | | √ | √ | √ | √ | | √ | √ | √ | | √ | √ |
| 9c | Changes in therapeutic intervention (with rationale) | | | | | √ | | | | √ | | | √ | | √ | | √ | √ |
| Follow-up and Outcomes | | | | | | | | | | | | | | | | | | |
| 10a | Clinician and patient-assessed outcomes (if available) . | | √ | √ | √ | √ | √ | √ | √ | √ | √ | √ | √ | √ | √ | √ | √ | √ |
| 10b | Important follow-up diagnostic and other test results | | | | | √ | √ | √ | √ | √ | √ | √ | √ | √ | √ | √ | √ | √ |
| 10c | Intervention adherence and tolerability (How was this assessed?) | | | | | √ | √ | √ | √ | √ | √ | | | √ | √ | | √ | √ |

| | | | | | | | | | | | | | | | | | | |
|---|---|---|---|---|---|---|---|---|---|---|---|---|---|---|---|---|---|---|
| 10d | Adverse and unanticipated events | | | | | √ | | | | √ | | √ | | | | | | √ |
| Discussion | | | | | | | | | | | | | | | | | | |
| 11a | A scientific discussion of the strengths and limitations associated with this case report | | | | | | | | √ | | | | | | √ | | √ | |
| 11b | Discussion of the relevant medical literature with references | √ | √ | √ | √ | √ | √ | √ | √ | √ | √ | √ | √ | √ | √ | √ | √ | √ |
| 11c | The scientific rationale for any conclusions (including assessment of possible causes) | √ | √ | √ | √ | √ | √ | √ | √ | √ | √ | √ | √ | √ | √ | √ | √ | √ |
| 11d | The primary “take-away” lessons of this case report (without references) in a one paragraph conclusion | √ | √ | √ | √ | √ | √ | √ | √ | √ | √ | √ | √ | √ | √ | √ | √ | √ |
| Patient Perspective | | | | | | | | | | | | | | | | | | |
| 12 | The patient should share their perspective in one to two paragraphs on the treatment(s) they received | | | | | | | | √ | | | | | | | | √ | √ |
| Informed Consent | | | | | | | | | | | | | | | | | | |
| 13 | Did the patient give informed consent? Please provide if requested | √ | √ | √ | √ | √ | √ | √ | √ | √ | √ | √ | √ | √ | √ | √ | √ | √ |
